# Pkd2 Affects Cilia Length and Impacts LR Flow Dynamics and *Dand5*

**DOI:** 10.3389/fcell.2021.624531

**Published:** 2021-04-01

**Authors:** Raquel Jacinto, Pedro Sampaio, Mónica Roxo-Rosa, Sara Pestana, Susana S. Lopes

**Affiliations:** CEDOC, Chronic Diseases Research Center, NOVA Medical School/Faculdade de Ciências Médicas, Universidade NOVA de Lisboa, Lisbon, Portugal

**Keywords:** left-right, cilia length, Pkd2, zebrafish, *dand5*, flow dynamics

## Abstract

The left-right (LR) field recognizes the importance of the mechanism involving the calcium permeable channel Polycystin-2. However, whether the early LR symmetry breaking mechanism is exclusively *via* Polycystin-2 has not been tested. For that purpose, we need to be able to isolate the effects of decreasing the levels of Pkd2 protein from any eventual effects on flow dynamics. Here we demonstrate that *curly-up* (*cup*) homozygous mutants have abnormal flow dynamics. In addition, we performed one cell stage Pkd2 knockdowns and LR organizer specific Pkd2 knockdowns and observed that both techniques resulted in shorter cilia length and abnormal flow dynamics. We conclude that Pkd2 reduction leads to LR defects that cannot be assigned exclusively to its putative role in mediating mechanosensation because indirectly, by modifying cell shape or decreasing cilia length, Pkd2 deficit affects LR flow dynamics.

## Introduction

Vertebrates establish left-right (LR) organization of the internal organs during development. This happens by the action of rotating cilia—5 μm hair-like structures on the surface of cells—in the left-right organizer (LRO) known as the node in mammalians and Kupffer’s vesicle (KV) in fish (Essner et al., 2005). These motile cilia generate a directional fluid flow from right to left, which leads to asymmetric intracellular calcium (Ca^2+^) oscillations (*icos*) (Yuan et al., 2015) in the first layers of cells surrounding the KV. Such *icos* were observed mainly on the left side of the KV midplane and were shown to be dependent on the presence of Pkd2 (Polycystin 2). Therefore, predominant left *icos* precede a left-biased asymmetric expression of genes (such as *nodal*, *lefty2*, and *pitx2*) in the lateral plate mesoderm. Subsequently, these genes will lead to asymmetric localization of internal organs.

The cation channel Pkd2 together with the mechanosensor Pkd1 (Polycystin 1) form a complex which, in the kidney, has the ability to sense the urine flow and induce an intracellular Ca^2+^ signal (Nauli et al., 2003). Pkd2 and its new partner Pkd1-like-1 have also been proposed as the mechanosensor-channel complex responsible for sensing the flow in the LROs of mice, medaka, and zebrafish and conveying the information into the adjacent tissues (Field et al., 2011; Kamura et al., 2011; Roxo-Rosa and Lopes, 2019). Despite working as a complex, it has been shown that decreasing Pkd2 alone is sufficient for a strong LR randomization both in mice and in zebrafish (Pennekamp et al., 2002; Bisgrove et al., 2005; Schottenfeld et al., 2007; Yoshiba et al., 2012).

However, how the flow is “perceived” by the LRO cells, remains controversial and has not been fully demonstrated. One explanation is provided by the “two-cilia hypothesis,” based on the fact that there are two types of cilia in the mouse node cells: one immotile, the sensory type, capable of detecting changes in the flow, and another motile rotating cilia generating the fluid flow movement (McGrath et al., 2003). An important finding was that Pkd2 absence in perinodal mouse cilia inhibits the usual asymmetric expression of cerberus-like2 (*cerl2*) on the right side, important for further LR asymmetry (Yoshiba et al., 2012). Motile and immotile cilia have also been found in the zebrafish KV (Sampaio et al., 2014). Perhaps more important, an unbalance on the ratio between motile and immotile cilia types leads to flow defects and to laterality problems with various degrees of severity (Tavares et al., 2017): *situs inversus*, a mirror image of the normal *situs* (*situs solitus*) or various abnormal combinations of thoracic and abdominal *situs*—heterotaxy—which is more severe and can even result in early death in humans (Fliegauf et al., 2007).

One of the advantages of using zebrafish to answer this question is the ability to manipulate cilia in early development and see the impact of each manipulation in LRO architecture, cilia motility, flow pattern, and organ *situs* in the exact same embryo. We previously characterized flow speed inside the KV by following native particles present in this organ (Sampaio et al., 2014). We reported that there is a main spot of faster flow in WT zebrafish in the anterior dorsal region of the LRO (Sampaio et al., 2014; Tavares et al., 2017) and that when this stereotyped flow pattern is perturbed the expression of the first asymmetric gene, *dand5*, becomes affected (Sampaio et al., 2014). As in mouse (Marques et al., 2004; Oki et al., 2009; Nakamura et al., 2012) and Xenopus (Schweickert et al., 2010), *dand5* starts to be symmetrically expressed and by a flow dependent process its expression decreases on the left side. This early *dand5* asymmetry is fundamental for generating the left sided *nodal* cascade of gene expression (Gourronc et al., 2007; Hojo et al., 2007; Lopes et al., 2010; Sampaio et al., 2014). Recently, Vick et al. (2018) showed in Xenopus that Pkd2 is upstream of Foxj1a and thereby affects cilia motility and LRO flow. In zebrafish, the role of Pkd2 has not been tested using adequate cilia or detailed flow quantification methods. In this study, we used both Pkd2 mutants and antisense technology to manipulate Pkd2 in the whole embryo or specifically in the LRO and investigated whether reducing Pkd2 levels affected LRO cilia length and motility thereby impacting on flow magnitude and pattern.

## Results

### LRO Fluid Flow Is Altered in Cup^–/–^ Mutants

First, we started by characterizing flow pattern using the zebrafish *pkd2* mutants, the *curly up* (cup) homozygous mutants (Schottenfeld et al., 2007). We previously demonstrated that *cup*^–^*^/^*^–^ embryos still have Pkd2 protein (Roxo-Rosa et al., 2015). Through immunostaining, we found Pkd2 protein present near the basal body and along the pronephric cilia from visually identified curly up tail *cup*^–/–^ mutants at 36 hpf (Roxo-Rosa et al., 2015). Since this mutant can only produce a truncated version of Pkd2 protein, which is not detected by our immunostainings, it is fair to assume that the functional protein detected must be maternally deposited as reported by Schottenfeld et al. (2007). So, we assume that embryos at KV stage still have Pkd2, even if in reduced levels. Knowing this, we set out to investigate the LRO flow speed and pattern of this “reduced Pkd2” condition.

**FIGURE 1:**
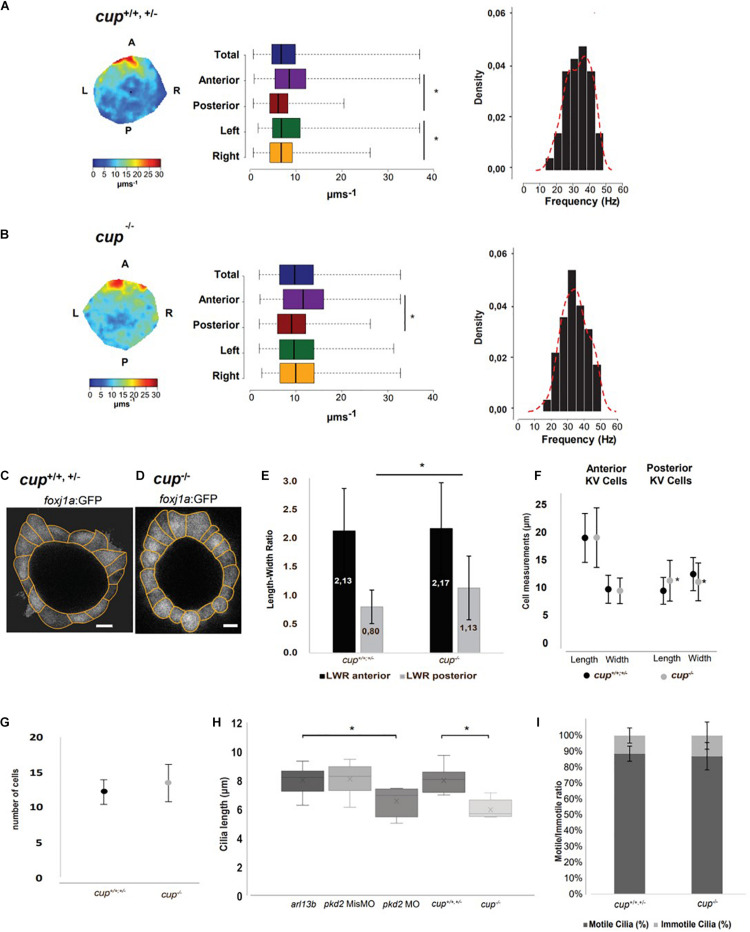
*cup*^–/–^ mutants have fluid flow defects in the LRO. **(A,B)** Fluid flow heatmap and quantification of *cup* siblings with straight tail (*n* = 8) and *cup*^–^*^/^*^–^ mutants with curly tail (*n* = 9), respectively. Asterisks represent statistical significance (Wilcoxon Test, *p*-value < 0.05). Cilia beat frequency (CBF) of *cup* siblings and *cup*^–/–^ mutants, respectively; *cup* siblings show an average CBF of 34.8 Hz and *cup*^–^*^/^*^–^ mutants an average of 34.4 Hz (paired *t*-test, *p*-value < 0.05). **(C,D)** Representative image of cell shapes from one KV from *cup* siblings and one KV from *cup*^–/–^ mutants, respectively. **(E)** Quantification of differences in length to width ratio and **(F)** differences in cellular length and width in *cup* siblings (*n* = 6) and *cup* mutants (*n* = 5); asterisks represent statistical significance (paired *t*-test, *p*-value < 0.05). **(G)** Number of cells present in *cup* mutants and sibling in the middle plane. **(H)** 3D cilia length measurements in live embryos injected with 50 pg of *arl13b-mCherry* mRNA (*arl13b*; *n* = 16) and injected with *pkd2* MisMO (*n* = 6), *pkd2* MO (*n* = 6), *cup* mutants (*n* = 8), and *cup* siblings (*n* = 13) with *arl13b-mCherry* mRNA; asterisks represent statistical significance (paired *t*-test, *p*-value < 0.05) **(I)** Motile/Immotile cilia ratio in the same *cup* mutants and *cup* siblings as in panel **(H)**. Scale bars 10 μm. L, left; R, right; A, anterior; P, posterior.

In order to evaluate the flow speed, we recorded the native particles inside the KV of multiple embryos at eight somite stage (ss) using high speed videomicroscopy as previously described (Sampaio et al., 2014). We then generated fluid flow heatmaps at 8 ss from the progeny of *cup* heterozygous crosses in a blind assay. After, we let these embryos develop until they presented either a straight tail that identified them as *cup*^±^ or *cup*^+^*^/^*^+^ (*n* = 8) or a curly tail that identified them as *cup*^–^*^/^*^–^ (*n* = 9). In this way, we found that flow speed from *cup*^–^*^/^*^–^ mutants was significantly increased. The flow quantification showed that *cup* homozygous mutants had faster flow (Wilcoxon test; *p*-value < 0.05) while still maintaining a similar spectrum of cilia beat frequencies (Figures 1A,B). The increased flow speed was unexpected and new as it had previously been noted that *pkd2* morphants had no problems in cilia (Bisgrove et al., 2005).

In search for a reason for the difference in fluid flow between siblings and *cup* homozygous mutants, we repeated the experiment in a *cup* mutant line raised in a *foxj1a*:GFP background to allow for visualization of KV cell shape and overall architecture (Figures 1C,D). The confocal images allowed detailed analysis of the midplane KV cells in two dimensions. Midplane was used because previous modeling studies by our lab (Sampaio et al., 2014) showed this plane displays a maximum lumen area and intercepts the flow vortex, being ideal for 2D studies. Results showed that while *cup*^–/–^ mutants still retained the anterior normal cell shape, with length to width ratio (LWR) > 1, the more posterior cells were significantly less wide compared to their siblings, showing a LWR proximate to 1, which indicates square cells (Figure 1E, *p*-value < 0.05). When analyzed in more detail, we found out that posterior KV cells in *cup*^–/–^ mutants had different length and width compared to those from their siblings (Figure 1F, *p*-value < 0.05). However, we did not find a difference in the average number of cells between mutants and their siblings (Figure 1G).

Next, we investigated cilia length and motility. To tackle this, we injected a minimal amount of *arl13b-mCherry* mRNA (50 pg) into 1-cell stage *cup* embryos and performed live imaging at 8 ss to measure cilia length in 3D and quantify cilia motility according to motile or immotile. After, embryos were dis-embedded and allowed to grow until they could be identified by the curly up tail phenotype. We saw a significant decrease of cilia length in *cup* mutants (mean of 5.99 μm) compared with *cup* siblings (8.02 μm, Figure 1H, *p*-value < 0.05). Since it is known that overexpression of *arl13b* leads to an increase on cilia length (Pintado et al., 2017), we also evaluated the impact of overexpressing *arl13b* mRNA in *pkd2* MO and *pkd2* MisMO injected embryos. Indeed, despite *pkd2* MO embryos were injected with *arl13b-mCherry* they still showed a significant reduction of cilia length while *pkd2* MisMO did not (Figure 1H, *p*-value < 0.05). Subsequently, continuing the search for an explanation for the increased flow speed in *cup* mutants we evaluated cilia motility and found that there was no significant difference in the motile to immotile ratio between *cup* siblings and mutants (Figure 1I).

In sum, differences in cell morphology found in *cup* mutants might affect the spacing between posterior KV cilia. However, more experiments should be done to further understand this phenomenon.

### LRO Targeted Pkd2 Knockdown Presents Slower Flow and Normal Fluid Flow Pattern

**FIGURE 2:**
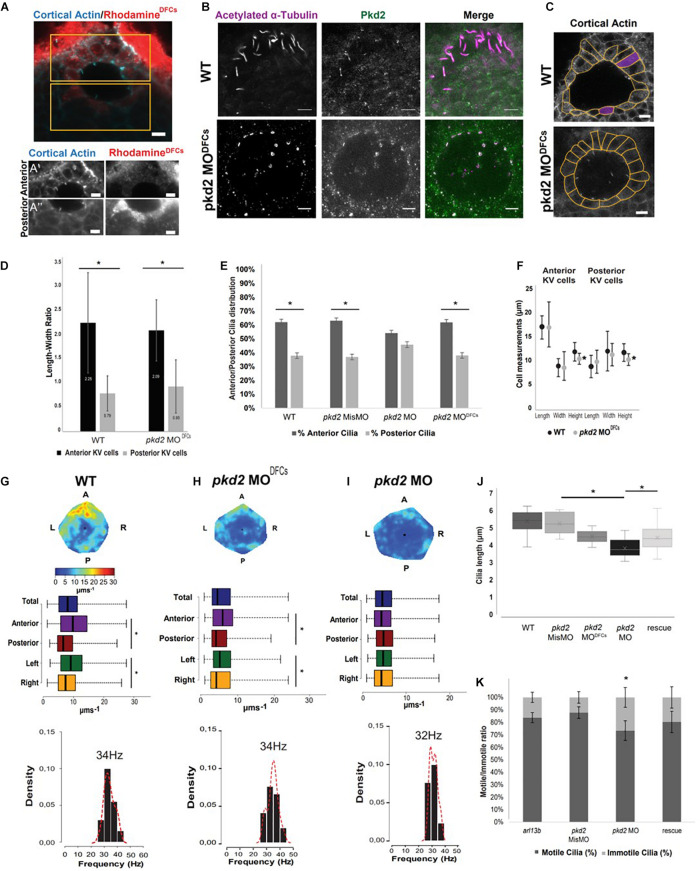
*pkd2* knockdown in the DFCs rescues KV fluid flow pattern. **(A)** Immunostaining for cortical actin and rhodamine showing the result of a successful injection of *pkd2* MO into DFCs, anterior **(A’)** and posterior **(A”)** panels in the different channels allow for better contrast/brightness balance; **(B)** Immunostaining for Pkd2 in WT and pkd2MO^DFCs^ embryos; **(C)** Representative images of KV architecture from one WT embryo and one *pkd2* MO^DFCs^ injected embryo. **(D)** Quantification of the differences in length to width ratio in the KV of WT (*n* = 6) and *pkd2* MO^DFCs^ (*n* = 9). **(E)** Cilia distribution in the antero-posterior axis of the KV of WT embryos (*n* = 22) and embryos injected with *pkd2* MisMO (*n* = 6), *pkd2* MO (*n* = 48) and *pkd2* MO^DFCs^ (*n* = 15). **(F)** Quantification of the differences in cellular length, width, and height, in the same WT and *pkd2* MO^DFCs^ injected embryos from panel **(D)**. **(G–I)** Fluid flow heatmap and quantification of WT (*n* = 8), *pkd2* MO^DFCs^ (*n* = 6) and *pkd2* MO 1-cell stage embryos (*n* = 7), respectively. Asterisks represent statistical significance (Wilcoxon Test, *p*-value < 0.05). **(J)** 3D cilia length measurement in WT (*n* = 23), *pkd2* MisMO (*n* = 8), *pkd2* MO 1-cell stage (*n* = 25), rescue (*n* = 19), and *pkd2* MO^DFCs^ (*n* = 10). **(K)** Motile/Immotile cilia ratio in live embryos injected with *arl13b-mCherry* mRNA (*n* = 8) and injected with *pkd2* MisMO (*n* = 10), *pkd2* MO (*n* = 6) and *pkd2* MO rescued with Xenopus *pkd2* mRNA (*n* = 5). Asterisks represent statistical significance (*p* < 0.05) with paired *t*-test. Scale bars 10 μm. L, left; R, right; A, anterior; P, posterior.

Since we wanted a better approach for Pkd2 removal, we next proceeded by knocking down *pkd2* by means of using an antisense morpholino oligonucleotide (MO), a method that better reduces Pkd2 levels in cilia (Roxo-Rosa et al., 2015) similarly to the PKD2 cilia deprived mouse model (Walker et al., 2019). However, we were already aware that blocking Pkd2 with this morpholino resulted in severely enlarged KVs, with architectural issues (Roxo-Rosa et al., 2015). Also, since it has already been reported that Pkd2 is present in many tissues, including notochord (Mangos et al., 2010), which in turn has been shown to play an important role in KV architecture (Compagnon et al., 2014), we decided to inject the *pkd2* MO in a later developmental stage and specifically target the precursors of the KV—the dorsal forerunner cells (DFCs) (Essner et al., 2005). By co-injecting the *pkd2* MO with the lineage tracer rhodamine-dextran, we could later select the embryos that only showed fluorescence in the yolk cell and the KV (Figure 2A). Below we shall refer to these embryos as *pkd2* MO^DFCs^.

Our first concern was to evaluate how strong was the Pkd2 knockdown with this DFCs approach. Unfortunately, even increasing the concentration of morpholino injected into DFCs, we were never able to completely remove Pkd2 from KV cilia (Figure 2B). Our second concern was to check if KV architecture was still compromised as in *cup*^–^*^/^*^–^ mutants. At first glance, *pkd2* MO^DFCs^ had a morphologically normal KV when compared with WT (Figure 2C). This was confirmed by measuring the Length-Width ratio of KV cells, which was similar in WT and pkd2 MO^DFCs^ (Figure 2D). This indicates that cells in the anterior part of the KV are preferentially taller making them more tightly packed together (LWR > 1), while cells in the posterior part are flatter (LWR < 1). This creates an asymmetry of cilia distribution across the anterior-posterior axis of the KV (60–40%), originating the anterior dorsal cluster—an important cilia cluster that creates the anterior flow hotspot (Wang et al., 2011, 2012). So, the asymmetry of cilia distribution is only lost in pkd2 MO due to the significant architecture alterations the KV suffers from lack of Pkd2 (Figure 2E). The recovery of the architecture with the DFCs-targeted injection of pkd2 MO shows a typical 60–40% distribution as seen in WT embryos (Figure 2E). Looking more carefully into KV architecture, we found a significant decrease in cell height between WT and embryos injected with 4 ng of *pkd2* MO^DFCs^ (Figure 2F; *p*-value < 0.05).

We then checked flow quantification and found that embryos injected with *pkd2* MO^DFCs^ had significant differences between anterior/posterior and left/right KV regions (Figure 2H), as in WT embryos (Figure 2G). These characteristics generated a heterogeneous flow map with a pattern similar to what is found in WT controls. However, total flow speed was slower compared to WT controls (Figure 2G WT 10 μms^–1^ vs. Figure 2H *pkd2* MO^DFCs^ 5 μms^–1^, *p*-value < 0.05), indicating that something else other than architecture, which was mostly unchanged, was impacting on flow speed. We also evaluated flow pattern in *pkd2* MO injected at 1 cell stage embryos and, as expected due to KV architectural problems already described by our lab, flow pattern was highly homogeneous (Figure 2I) and also very slow (Figure 2I, pkd2 MO 4 μms^–1^, *p*-value < 0.05). It also had a significantly slower cilia beat frequency (32 Hz compared with WT 34 Hz, *p*-value < 0.05). Next, we tested for cilia length upon *pkd2* manipulation. Our results clearly showed that cilia length was shorter compared to the controls, either for injections of *pkd2* MO at 1-cell stage or for *pkd2* MO^DFCs^ (Figure 2J, *p*-value < 0.05). A partial rescue was obtained by injecting 1,000 pg of *pkd2* mRNA (Figure 2J, *p*-value < 0.05). Cilia length, this time measured in fixed samples without arl13b OE was evaluated by anti-acetylated alpha-tubulin staining explaining the general lower values in Figure 2J compared to Figure 1H. We next evaluated cilia motility and found a significant reduction of cilia motility in *pkd2* morphants (Figure 2K, *p*-value < 0.05), which was also rescued by the injection of *pkd2* mRNA.

In sum, *pkd2* MO impacted extensively on KV architecture, reduced cilia length and motility, which resulted in a slow and homogenous flow. DFCs-targeted injection of *pkd2* MO was less disruptive for KV architecture, but still impacted on cilia length. The resulting flow was heterogeneous but significantly slowed.

### Impairment of Pkd2 and Fluid Flow Similarly Affect *dand5* Expression Pattern and Organ Situs

**FIGURE 3:**
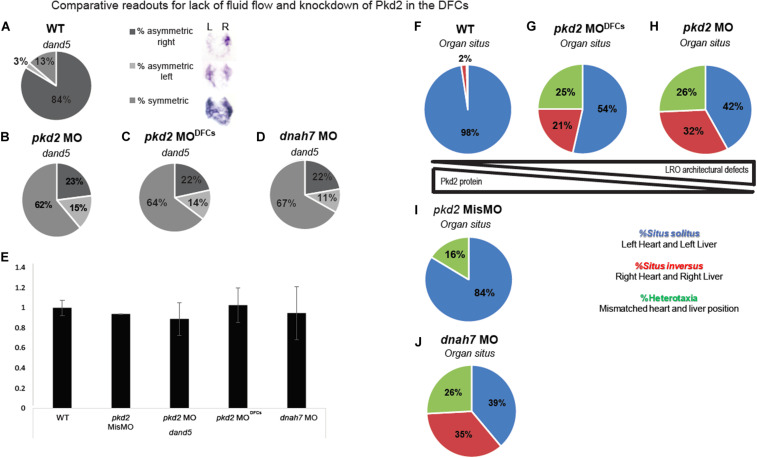
Comparative readouts for lack of fluid flow and knockdown of Pkd2 in the DFCs **(A–D)** *dand5* expression pattern quantification by *in situ* hybridization of WT embryos, *pkd2* MO, *pkd2* MO^DFCs^, and *dnah7* MO. **(E)** *dand5* expression level in fold change quantified by qRT-PCR; asterisks represent statistical significance (paired *t*-test, *p*-value < 0.05). **(F–J)** Organ *situs* quantification by scoring heart and liver laterality in the same larvae in WT, *pkd2* MO^DFCs^, *pkd2* MO 1-cell stage, *pkd2* Mismatch MO and *dnah7* MO.

In this new comparative experiment, we used two readouts: (a) *dand5* expression, a nodal inhibitor known to be the first asymmetric expressed gene during the LR axis establishment and (b) internal organ *situs*. In WT zebrafish embryos, *dand5* is mainly expressed on the right side from eight somite stage onward (Lopes et al., 2010; Figure 3A). Our results showed that both manipulations of Pkd2 (whole-embryo and DFCs-injected) lead to *dand5* becoming predominantly symmetric (Figures 3B,C). Similarly, when flow is abrogated by rendering all cilia immotile (*dnah7* MO) (as in Sampaio et al., 2014), *dand5* also became predominantly symmetric (Figure 3D). This shows that, in terms of expression pattern, *dand5* becomes highly symmetric both when there is “no flow” (67%) and when there is “reduced Pkd2 level” (64%). This fact suggests that a Pkd2-mediated sensing of flow is very important to define *dand5* expression pattern, which in WT may be accomplished by higher degradation rates of *dand5* mRNA on the left side, where flow is more strongly detected (Oki et al., 2009; Schweickert et al., 2010; Nakamura et al., 2012; Sampaio et al., 2014). As for mRNA expression quantification of *dand5*, qRT-PCRs were performed for each condition. For all treatments there were no differences between WT, *pkd2* MO^DFCs^, *pkd2* mismatch MO and *dnah7* MO (Figure 3E). This suggests that both impaired flow and manipulation of Pkd2 do not have an impact on *dand5* transcription.

Lastly, we analyzed organ *situs*. Controls showed most cases (97.5%) with left heart and left liver, which means *situs solitus* in zebrafish (Figure 3F). All treatments showed a strong and significant reduction of *situs solitus* when compared to the control situation (Figure 3F) and to the *pkd2* mismatch MO^DFCs^ (Figure 3I) (Fisher test with Bonferroni correction, *p*-value < 0,0125). Both *pkd2* manipulations lead to different amounts of Pkd2 protein being present in the LRO and adjacent tissues, which impacts in LRO architecture and flow pattern/strength defects. However, for LR axis establishment, there seems to be no difference between having “no flow” (Figure 3J) or having defective LRO architecture together with defective flow pattern due to reduced levels of Pkd2 (Figures 3G,H). In sum, *dand5* fails to become asymmetric and organ *situs* becomes randomized between normal (*situs solitus*), a total inversion of *situs* (*situs inversus*) or mismatched combinations of heart and liver position (heterotaxia).

## Discussion

In the field of LR axis determination there is increasing evidence for exciting biophysical interactions between extracellular fluid dynamics and cellular sensory mechanisms (Supatto and Vermot, 2011; Yoshiba et al., 2012; Sampaio et al., 2014; Smith et al., 2014). With this study we can further confirm that Pkd2 channel plays a major role in the LR axis. Here we use a previously tested antisense morpholino for Pkd2 (Schottenfeld et al., 2007), that effectively reduces Pkd2 levels by antibody staining and leaves only a ring of expression around the base of the cilia (Roxo-Rosa et al., 2015), as described by Walker et al. (2019) for a cilia deprived Pkd2 mouse model. We showed that reducing Pkd2 levels by knockdown experiments decreased cilia length and consequently diminished LRO flow speed, triggering early and late LR defects, as shown by *dand5* symmetric expression pattern and randomized organ *situs*, respectively.

Cilia length was known to greatly affect flow production as the flow produced by an individual cilium scales with the cube of cilia length (Sampaio et al., 2014; Smith et al., 2014). Additionally, we showed here that reduced Pkd2 levels compromised *dand5* mRNA degradation, while its transcription was not quantitatively affected (Figure 3). Furthermore, the differences on *dand5* mRNA patterns between knocking down *dnah7 or pkd2* were not statistically significant (Figure 3). However, if we could separate the effect of Pkd2 on flow dynamics from its postulated role in mechanosensation we would predict a different scenario, with near 100% of *dand5* symmetric expression.

As in the Xenopus model (Vick et al., 2018) this current study reveals that it is not possible to reduce Pkd2 levels without affecting LRO fluid flow. However, in zebrafish Pkd2 morphants the reasons are different as the number of cilia is not affected (Roxo-Rosa et al., 2015) nor *foxj1a* expression is changed (unpublished data). The reason for the decreased cilia length is not yet understood. However, it is important to disclose this phenotype as it affects the generation of flow. So, comparative studies between animal models within the LR field need to be aware that; 1) Pkd2 in the zebrafish embryo is maternally deposited (Schottenfeld et al., 2007); 2) that even when effectively knocked down, *pkd2* affects other variables, such as cilia length and flow dynamics as well as LRO architecture and fluid volume (Roxo-Rosa et al., 2015), factors that are all crucial in LR early establishment at the LRO level.

We hope this study raises attention to what extent we can use the zebrafish model to study Pkd2 mechanisms, as we do not know the order of events, for example if cell shape changes result from abnormal mechanosensation, or if it is the other way around. In addition, from a slight different angle, we do not know if shorter cilia are a response to cell shape changes or their potential cause.

Regarding the contradictory increase in flow speed scored in *cup* homozygous mutants, we discarded a potential increase in motile cilia or in CBF and confirmed that these mutants also display reduced cilia length. Therefore our data points to cell morphology as the only measured parameter that could cause the referred flow increase. Nevertheless, we cannot assess causality without at least generating appropriate numerical simulations. As to the upstream cause of these differences between mutants and knockdowns we also cannot exclude that the mutation in *cup*^–^*^/^*^–^ mutants, forms a truncated protein that is not degraded and may have undetermined functions and consequences.

Our study intends to alert for some Pkd2 zebrafish facts at the same time it exposes missing causal links. Pkd2 is a central protein in LR development that needs to be studied using conditional mutants with full loss of function in the tissues and in the cilia from those tissues being studied, as reported for the mouse model in some instances (Yoshiba et al., 2012; Walker et al., 2019).

### Experimental Procedures

#### Fish Stocks and Genetics

The following zebrafish lines were maintained and used as described elsewhere (Westerfield, 2000): wild-type (AB), *Tg(foxj1a:GFP)* (Caron et al., 2012) and cup[± ;*Tg(foxj1a:GFP*)]^tc321^. Embryos were raised at 28 or 32°C, depending on the experiment, in E3 embryo media and staged accordingly (Kimmel et al., 1995). Procedures with zebrafish were approved by the Portuguese DGAV (Direcção Geral de Alimentação e Veterinária).

#### Injections of Morpholino Oligonuclotides

*dnah7* morpholino (Sampaio et al., 2014) was diluted in sterile water and injected at one cell stage at a dose of 3 ng per embryo for *dnah7*. Pkd2 morpholino injection was diluted in sterile water and injected at one cell stage at a dose of 2.5 ng per embryo. To generate chimeric *pkd2* knockdown in DFCs, *pkd2* morpholino (Schottenfeld et al., 2007) was diluted in 10,000 MWt rhodamine-dextran solution (1:4; Sigma-Aldrich) and injected at a dose of 4.2 ng per embryo into the yolk of 512–1,000-cell-stage embryos as previously described (Amack and Yost, 2004). Morpholino injection efficiency was thoroughly controlled as follows: specific *pkd2* MO targeting to DFCs was determined by the rhodamine lineage tracer in KV and yolk cells of the selected embryos (Amack and Yost, 2004; Figure 2A); embryos injected with *dnah7* morpholino oligonucleotide were carefully screened by high speed-videomicroscopy for confirming cilia immotility throughout the entire KV. A mismatch *pkd2* morpholino was injected in the same conditions (4.2 ng per embryo) as control. 1,000 pg of Xenopus *pkd2* mRNA was injected at 1 cell stage alone and in combination with *pkd2* MO for rescue experiments. To assess cilia motility and length live, 50 pg of *arl13b-mCherry* mRNA was injected into 1 cell stage embryos.

#### Live Imaging for Flow Recording

Mounted embryos between 13–14 hpf were set under the 100x/1.30 NA oil immersion objective lens on a Nikon Eclipse Ti-U inverted microscope at room temperature (26°C). All images were taken with the dorsal roof of the KV facing the objective lens. Bright field images were recorded with a FASTCAM MC2camera (Photron Europe, Limited) controlled with PFV (Photron FASTCAM Viewer) software. Native KV particles were filmed at 60 fps for 30 s while cilia were recorded at 500 fps for 2 s. KV flow and CBF measurements were analyzed using Fiji software as described previously (Sampaio et al., 2014). We have successfully analyzed eight WT embryos, nine cup^–/–^ embryos, and eight cup^±^/cup^+/+^ siblings, four *dnah7* MO injected embryos, seven *pkd2* MO 1-cell stage injected embryos and six *pkd2* MO^DFCs^ injected embryos.

#### Immunofluorescence and in *in situ* Hybridization

Whole-mount immunostaining and *in situ* hybridization were performed as described previously (Lopes et al., 2010). Antibodies used for immunostaining were anti-acetylated alpha-tubulin (1:400; Sigma), Alexa Fluor 488 (Invitrogen; 1:500) and Alexa fluor 488 phalloidin (Invitrogen/molecular probes 1:100). Pkd2 immunostaining was performed as described in Roxo-Rosa et al., 2015. Individual *dand5 in situ* hybridizations were performed at 8–10 somite stage in 37 WT embryos, 13 *pkd2* MO 1-cell stage, 14 *pkd2* MO^DFCs^ and nine *dnah7* MO. *foxa3 in situ* hybridizations at 53 hpf were performed as described elsewhere (Thisse and Thisse, 2008). Gene expression and *situs* scoring were performed double blind by two investigators and the results were analyzed by Fisher’s exact test with Bonferroni correction for multiple comparisons and by Binomial exact test for comparisons in the same genetic background.

#### Cell Shape and Length-Width Ratio Measurements

Cup[±;*Tg(foxj1a:GFP*)]^tc321^ embryos were mounted live in 2% (w/v) agarose and covered with E3 medium for confocal epifluorescence microscopy live imaging at room temperature. To assess cell shape, whole KVs were scanned with *z* sections of 0.5 μm, with an acquisition rate of less than 1 fps. After acquisition, embryos were retrieved from the agarose and let develop for heart scoring and tail phenotype. We imaged 12 *cup*^–/–^ embryos and 19 *cup* sibling embryos. Embryos injected with *pkd2* MO^DFCs^ were immunostained at 14 hpf for actin cytoskeleton and mounted in PBS 1X for confocal epifluorescence microscopy in the same conditions. We imaged 15 *pkd2* MO^DFCs^ injected embryos and 15 siblings from the same fish lines. Selected stacks were subsequently analyzed in Amira for 3D cell shape in KV midplane (five to six embryos for each condition). Results were statistically analyzed by using the paired *t*-test and statistical significance was set at *p*-value < 0.05.

#### Cilia Length and Motility

Live embryos were injected at 1 cell stage with 50 pg of *arl13b-mCherry* and then later mounted in 1% (w/v) low-melting agarose at 8 ss, covered in E3 medium. Live imaging was performed in a Zeiss LSM 710 confocal microscope with an Olympus 40× water immersion lens (NA 0.8) at room temperature. 3D length of motile and immotile cilia was quantified based on the radial fluorescence intensity profile of each cilium, through semiautomated detection in IMARIS software program (Bitplane, United Kingdom). A total number of 16 *arl13b-mCherry* embryos (291 cilia), six *arl13-mCherry* + *pkd2* MisMO embryos (92 cilia), six *arl13b-mCherry* + *pkd2* MO (94 cilia), eight *arl13b-mCherry cup* mutants (117 cilia), and 13 *arl13b-mCherry cup* siblings (257 cilia) were measured at 8 ss.

To assess motility, KVs were scanned with *z* sections of 0.5 μm, with an acquisition rate of 9.6 slices per minute (6.25 s per slice), which provided a pixel dwell time of 22.4 μs. A total number of eight a*rl13b-mCherry*, six *arl13b-mCherry* + *pkd2* MO, six *arl13b-mCherry* + Xenopus *pkd2* mRNA + *pkd2* MO (rescue), and 10 *arl13b-mCherry* + *pkd2* MisMO were measured at 8 ss.

Fixed embryos were immunostained with anti-acetylated α-tubulin and imaged in the same confocal. 3D cilia length was measured using the “Simple Neurite Tracer” plugin in Fiji (Longair et al., 2011). A total of 22 WT embryos (1,127 cilia), eight *pkd2* MisMO (497 cilia), 25 *pkd2* MO (1,592 cilia), 19 *pkd2* MO, and Xenopus *pkd2* mRNA (rescue; 996 cilia) and 10 *pkd2* MO^DFCs^ (379 cilia) were measured at 8–10 ss.

#### Heart and Gut Laterality

At 30 hpf we evaluated heart jogging using a stereoscopic zoom microscope (SMZ745, Nikon Corporation) to observe the embryos from the ventral side. These embryos were then allowed to develop in separated petri dishes and at 53 hpf, embryos were fixed and processed for *foxa3 in situ* hybridizations to assess gut laterality. We could then pair the heart *situs* with gut *situs* for each treatment and attribute an embryo *situs*. We scored organ *situs* in 159 WT, 31 *dnah7* MO embryos, 140 *pkd2* MO 1-cell stage embryos, 56 *pkd2* MO^DFCs^ embryos and 31 *pkd2* mismatch control-MO injected embryos.

#### Quantitative PCR

Four groups of ten embryos were used; one group for untreated (control) and the others were injected as explained above and let develop until 8–10 somite stage. After thorough scoring of rhodamin expression only in KV and yolk cell and complete cilia immotility, total RNA was extracted using the Qiagen RNeasy Mini Kit (ref number 74104) and reverse transcribed using both oligo(dT)_18_ and random hexamer primers with the RevertAid First Strand cDNA Synthesis Kit (ref number K1622) following the manufacturers’ instructions. This was repeated three times for three different biological replicates. Expression was quantified by PCR using Roche SYBR Green I Master (reference number 04887352001) and run in a Roche LightCycler^®^ 96 Real-Time PCR System. Results were analyzed and depicted as fold-change of transcript levels in injected embryos relative to transcript levels in control embryos. The *p*-value represents significance in the pairwise comparison of transcript levels between injected and control embryos as determined using the paired *t*-test. Statistical significance was set at *p*-value < 0.05. *dand5* levels were normalized in relation to *eukaryotic elongation factor 1 alpha 1 like 1* (*eef1al1*) and *ribosomal protein L13a* (*rpl13a*) expression. Primer sequences used were as follows: *dand5* forward 5′-CCGCAATCCTGACCCATAGCAA-3′ and reverse 5′-CTCCTCCGTTATGCGCTGTGTA-3′; *eef1al1* forward 5′-CCTTCAAGTACGCCTGGGTGTT-3′ and reverse 5′-CACAGCACAGTCAGCCTGAGAA-3′; *rpl13a* forward 5′-T GACAAGAGAAAGCGCATGGTT-3′ and reverse 5′-GCCTG GTACTTCCAGCCAACTT-3′.

## Data Availability Statement

The raw data supporting the conclusions of this article will be made available by the authors, without undue reservation.

## Ethics Statement

The animal study was reviewed and approved by Direção Geral Alimentação e Veterinária.

## Author Contributions

SL, RJ, and PS conceived and designed the experiments. SL, RJ, and PS analyzed the data and wrote the manuscript. RJ, PS, SP, and MR-R performed the experiments. All authors revised the manuscript.

## Conflict of Interest

The authors declare that the research was conducted in the absence of any commercial or financial relationships that could be construed as a potential conflict of interest.
